# Evaluating clinic and community-based lifestyle interventions for obesity reduction in a low-income Latino neighborhood: Vivamos Activos Fair Oaks Program

**DOI:** 10.1186/1471-2458-11-98

**Published:** 2011-02-14

**Authors:** Rebecca L Drieling, Jun Ma, Randall S Stafford

**Affiliations:** 1 Program on Prevention Outcomes and Practices, Stanford Prevention Research Center, Stanford School of Medicine, Stanford, CA, USA; 2 Palo Alto Medical Foundation Research Institute, Palo Alto, CA, USA

## Abstract

**Background:**

Obesity exerts an enormous health impact through its effect on coronary heart disease and its risk factors. Primary care-based and community-based intensive lifestyle counseling may effectively promote weight loss. There has been limited implementation and evaluation of these strategies, particularly the added benefit of community-based intervention, in low-income Latino populations.

**Design:**

The Vivamos Activos Fair Oaks project is a randomized clinical trial designed to evaluate the clinical and cost-effectiveness of two obesity reduction lifestyle interventions: clinic-based intensive lifestyle counseling, either alone (n = 80) or combined with community health worker support (n = 80), in comparison to usual primary care (n = 40). Clinic-based counseling consists of 15 group and four individual lifestyle counseling sessions provided by health educators targeting behavior change in physical activity and dietary practices. Community health worker support includes seven home visits aimed at practical implementation of weight loss strategies within the person's home and neighborhood. The interventions use a framework based on Social Cognitive Theory, the Transtheoretical Model of behavior change, and techniques from previously tested lifestyle interventions. Application of the framework was culturally tailored based on past interventions in the same community and elsewhere, as well as a community needs and assets assessment. The interventions include a 12-month intensive phase followed by a 12-month maintenance phase. Participants are obese Spanish-speaking adults with at least one cardiovascular risk factor recruited from a community health center in a low-income neighborhood of San Mateo County, California. Follow-up assessments occur at 6, 12, and 24 months for the primary outcome of percent change in body mass index at 24 months. Secondary outcomes include specific cardiovascular risk factors, particularly blood pressure and fasting glucose levels.

**Discussion and Conclusions:**

If successful, this study will provide evidence for broad implementation of obesity interventions in minority populations and guidance about the selection of strategies involving clinic-based case management and community-based community health worker support.

**Clinical Trial Registration:**

ClinicalTrials.gov: NCT01242683

## Background

### Obesity-related health and economic burden

There is a pressing need for obesity management strategies to address the growing prevalence of excess weight in the U.S. With 68% of adults overweight (34%) or obese (34%), [1-3] obesity-attributable medical costs in the U.S. average $147 billion per year and account for almost 10% of the total annual medical expenditures[4]. Obesity is strongly associated with higher rates of coronary heart disease (CHD), stroke, and shorter life expectancy, and CHD risk factors (e.g., diabetes and hypertension)[5]. Three-quarters of obese Americans have at least one CHD risk factor reversible through weight loss[6]. Fortunately, even modest, 5-10% reductions in body weight, as opposed to achieving ideal weight, are associated with clinically significant improvements in CHD risk factors[7,8]. Persons of low socioeconomic status (SES) are disproportionately affected by the CHD risk from obesity, in part, because they are less likely to receive adequate clinical care[9,10]. Persons of low SES also face more environmental factors associated with obesity, such as lack of access to healthy foods, high prevalence of high-calorie low-nutrient foods, and limited safe places to exercise[11-15].

### Benefits of lifestyle interventions

Intensive lifestyle interventions focused on nutrition and physical activity may effectively promote lifestyle changes that reduce weight and other CHD risk factors,[16,17] but have often not been implemented and evaluated in the low SES communities that most need them. Convincing studies, including the Stanford Heart to Heart Project and the Diabetes Prevention Program, have shown efficacy of intensive individualized lifestyle counseling for sustained weight loss and CHD risk factor reduction[18-23]. Research also shows that lifestyle counseling in groups may be as effective for achieving weight loss[24-26] and more economical compared to individual counseling[25,27]. However, clinic-based lifestyle interventions for weight loss may not sufficiently address environmental barriers in low SES communities[12-15].

Given the profound impact neighborhood characteristics have on weight [28-30], clinic-based lifestyle interventions for weight loss may be enhanced by support in the patient's social environment. A growing body of research suggests that community health workers (CHWs) can connect clinic-based CHD risk management services and the social environment for persons of low SES[31-33]. CHWs are members of local communities who help community members utilize neighborhood resources and develop community resilience. CHWs with training in health care can provide support to extend practical applications of primary care and lifestyle interventions into homes and neighborhoods and fill gaps in clinic-based programs, especially around access to resources[32-34].

Despite growing evidence about the effectiveness of intensive lifestyle counseling[18-23] and benefit of CHWs for weight loss and CHD risk factor reduction [31,32,35], few studies compare the benefit of CHW support to clinic-based obesity-reduction interventions[35]. Reducing the enormous clinical consequences of obesity demands that our health care system resolutely integrate the mission of obesity management into clinic-based primary care and community-based programs, particularly in low SES communities. Targeting upstream health behaviors, such as nutrition and physical activity choices, has the potential to reduce adverse obesity-related disease and its economic impacts.

### Aims

Vivamos Activos Fair Oaks (VAFO) will evaluate the clinical and cost-effectiveness of two lifestyle interventions to reduce body weight among obese Latinos of low SES who are patients of a county health clinic and have additional CHD risk factors. The first intervention provides intensive group and individual lifestyle counseling and case management for weight reduction and is provided by health educators in a primary care clinic (CM arm). The second intervention combines the health educator intervention with CHW support for nutrition and physical activity in participant's home and community (CM+CHW arm). Furthermore, the study aims to compare the two interventions with usual care (UC) and then transition the favored intervention to a sustainable community health program. The study builds on national model programs of successful weight loss, particularly DPP. Implementation also relies on our previous Stanford Heart to Heart clinical trial, the primary care services in the Fair Oaks Clinic of San Mateo, and the community programs developed by El Concilio of San Mateo County[23,36,37].

## Methods

### Study design

The VAFO is a randomized clinical trial in which obese adults with CHD risk are randomized to one of three study arms: UC, CM, or CM+CHW. All study procedures and materials were approved by the Stanford Institutional Review Board (IRB) and an independent IRB serving San Mateo Medical Centers (SMMC).

### Study setting

The VAFO project is set in the North Fair Oaks neighborhood of San Mateo County and conducted out of the Fair Oaks Clinic, an adult health clinic within the SMMC system. The North Fair Oaks neighborhood is an unincorporated 1.2 square mile trapezoidal area surrounded by more affluent cities. Fair Oaks has a population of 15,400 people, of which 69% is Latino and 50% were born outside the U.S[38]. SMMC provides medical service to most low-income residents of the County, but has 35% fewer physicians per 100,000 capita than the national average[39]. The shortage of physicians disproportionately affects local Hispanics, 34% of which earn below 200% of the federal poverty line and rely on county health services[40]. In 2008, 57% of adult San Mateo County residents were overweight (19% obese) and 85% had at least one CHD risk factor[40].

The Fair Oaks neighborhood was chosen because of the high prevalence of low-income Hispanics and a prior partnership between our research group and key stakeholders, including the Fair Oaks Clinic and El Concilio of San Mateo County. Fair Oaks Clinic provides primary care, mental health counseling, and social services. El Concilio of San Mateo County, a community-based organization, provides nutrition, physical activity, and chronic disease management programs and trains CHWs. In particular, El Concilio operates a diabetes and metabolic syndrome management program in conjunction with the Fair Oaks Clinic.

### Eligibility criteria

Obese Spanish-speaking patients age 18 and older with at least one conventional CHD risk factor (i.e. diabetes, elevated fasting glucose or hemoglobin A1c levels, high blood pressure, elevated lipid levels) are eligible to join the study. Exclusion criteria are designed to: 1) minimize safety concerns; 2) prevent loss to follow-up; and 3) avoid potential contamination between study arms (Table 1).

**Table 1:** Participant inclusion and exclusion criteria

Inclusion criteria	
1.	Age 18 years or older;
2.	Body mass between 30 and 55 kg/m^2^;
3.	One or more CHD risk factors:
	a) Systolic blood pressure between 130 and 200 mmHg;
	b) Diastolic blood pressure between 80 and 105 mmHg;
	c) Total cholesterol > 180 mg/dL;
	d) LDL cholesterol > 120 mg/dL;
	e) HDL Cholesterol < 40 mg/dL;
	f) Triglycerides > 150 mg/dL;
	g) HbA1c between 6.0 and 11.5%;
	h) Fasting plasma glucose between 95 and 400 mg/dL;
	i) Diagnosis of Type 2 diabetes;
4.	Residing in catchment area of the Fair Oaks Clinic and receiving primary care at Fair Oaks Clinic.
**Exclusion criteria**	

1.	Inability to speak Spanish;
2.	Unwilling to attempt weight loss;
3.	Significant medical co-morbidities, including uncontrolled metabolic disorders (e.g., thyroid, diabetes, renal, liver), unstable heart disease, advanced heart failure, and ongoing substance abuse;
4.	Taking more than 10 prescription medications;
5.	Psychiatric disorders requiring antipsychotics or multiple medications;
6.	Body weight change > 25 lbs. in the preceding 3 months;
7.	Pregnant, planning to become pregnant, or lactating less than six months;
8.	Family household member already enrolled in the study;
9.	Current or planned participation in a study that would limit full participation in VAFO;
10.	Refusal of home visits by study staff;
11.	Resident of a long term care facility;
12.	Plans to move during the study period (24 months post-randomization);
13.	Investigator discretion for clinical safety or adherence reasons (e.g., unstable housing,
	chronic pain that impedes physical activity).

### Recruitment and screening

The target sample size for VAFO is 200 participants randomized during a 15-month period. Participants are recruited for screening by study staff who solicits patients in the clinic or by referral from a primary care provider (PCP). After obtaining PCP approval for medical appropriateness, chart review and telephone screening are performed to assess basic eligibility criteria. Those not excluded receive formal eligibility determination at clinical baseline visits (BV1 and BV2) which both occur within three weeks of randomization. BV1 includes assessment of biomedical eligibility, administration of survey questionnaires, and receipt of a pedometer to track physical activity. BV2 occurs seven to ten days after BV1 and includes review of pedometer data and a fasting blood draw. Following standard human subjects protocol, all participants provide informed consent during the screening process.

### Randomization and blinding

Participants are randomized to one of three arms according to the ratio 1 UC: 2 CM: 2 CM+CHW. After all BV2 data are collected, a blinded data analyst confirms study data completion and randomizes the participant to one of the three arms in permuted blocks stratified by sex, BMI (30-34.9, 35-39.9, or ± 40), and diabetes status. Follow-up data collection (6, 12, 18, and 24 months) is performed by data collectors blinded to intervention status.

### Baseline and follow-up measures and data collection

Participant time commitment for all research-related measurements is approximately 2.5 hours at baseline (for telephone screening, BV1, and BV2 combined), 2 hours each for follow-up clinic visits (6, 12, and 24 month), and 45 minutes for the 18-month telephone assessment (Table 2).

**Table 2:** List of study measures and data collection schedule

		Follow-up month			
	**Baseline**	**6**	**12**	18*	**24**

**Clinical Measures**					

Height	X				
Weight	X	X	X		X
Waist circumference	X		X		X
Blood pressure	X	X	X		X
Fasting blood: Total cholesterol, LDL-C, HDL-C, triglycerides, glucose, HBA1c	X	X	X		X
C-Reactive Protein	X		X		X
**Questionnaires**					

Physical Activity Readiness Survey	X				
Demographic history	X				
Employment, Income	X	X	X	X	X
Modified BRFSS exercise questions	X	X	X	X	X
Block Brief food Questionnaire	X	X	X		X
6-item food security assessment	X	X	X	X	X
Depression Survey (CESD)	X	X	X	X	X
Obesity Related Problems Scale	X	X	X	X	X
Pittsburgh Sleep Quality Index			X		X
Strength of Religious Faith Questionnaire			X		X
Researcher designed physical activity and nutrition questions	X	X	X	X	X
Smoking	X	X	X	X	X
Adverse events		X	X	X	X
Medication use	X	X	X	X	X
**Pedometer**					

7 day pedometer log	X	X	X		X
**Data extracted from electronic SMMC medical records system**					

Healthcare utilization (hospitalizations, emergency room and outpatient visits)	X	X	X		X
Medications prescribed	X	X	X		X

*18-month visit is conducted by telephone.					

### Primary and secondary outcomes

Primary hypotheses will be tested based on change in BMI change from baseline to 24 months. Data collected at the interim time points will help assess effects of intensive phase of intervention (baseline to 12 months) and intervention durability throughout the maintenance phase (12 month to 24 months). The primary outcome is change in BMI calculated as kg/m^2^. Weight is collected at BV1 and each follow-up clinic visit using the average of two readings from a digital scale. Height is collected at BV1 using the average of two readings from a wall-mounted stadiometer. Weight and height are both collected from participants in light indoor clothes without shoes.

Secondary outcomes measure obesity-related biomedical cardiovascular risk factors. Plasma lipids, glucose, hemoglobin A1c, and C-reactive protein are collected after an overnight fast. Waist circumference is collected with a tape measure around the waist at the midpoint between the lowest part of the ribcage and the top of the pelvic bone. Resting blood pressure is measured after the patient has sat quietly with feet flat on the floor for five minutes. Three blood pressures are obtained on alternating arms and the mean of the second and third readings is used in analysis.

Additional secondary outcome measures include behavioral and psychosocial factors that might moderate the intervention effect. The Obesity Related Problems Scale is administered to understand impacts of obesity on social beliefs and attitudes[41]. The Center for Epidemiologic Studies-Depression Scale is administered to assess prevalence and change in depression[42]. The 6-item Food Security Assessment is administered to assess use of food assistance and ability to purchase food[43]. The Pittsburgh Sleep Quality Index [44] and the Strength of Religious Faith Questionnaire are also administered[45]. Additional questions designed by the study team assess neighborhood safety and social support networks.

Physical activity levels are measured at each clinical assessment point by recording seven days of steps using a pedometer worn by participants and by interviewer-administered physical activity questions adapted from the Centers for Disease Control Behavioral Risk Factor Surveillance System[46]. Dietary intake is assessed by administering the Block Brief Food Questionnaire[47]. Additional physical activity and dietary patterns are assessed by administering questions designed by the study researchers about sedentary activities, fast food consumption, and use of neighborhood physical activity and nutrition resources. Socio-demographic information is collected including education level, place of birth, language spoken at home, employment status and income. Self-reported and medical record data on hospitalizations, emergency room and outpatient visits and prescriptions are assessed for impact on medical resource utilization.

### Process measures

Reasons for joining and not joining the study are used to evaluate recruitment techniques. Attendance of friends and relatives at intervention visits is assessed to evaluate inclusion of extended social networks. Self-monitoring forms are collected to assess use of self-regulation tools. Additionally, key informant interviews will be conducted throughout the intervention period to evaluate how the VAFO coordinates with other clinic services and to refine the program for dissemination.

### Interventions

#### Theoretical basis

The overriding theoretical framework for the intervention is derived from Social Cognitive Theory (SCT)[48] and the Transtheoretical Model (TTM) of Behavior Change. SCT emphasizes the reciprocal determinism between individual, environment, and behavior. TTM recognizes that behavior change is a dynamic process that moves through stages of readiness to change a problem or maintain positive or healthy behaviors. SCT assumes that behavior change is more likely with increased behavior capability, which is strengthened through skill building and self-regulation. Equally important are confidence in performing a given behavior individually (self-efficacy) and with a support group (collective efficacy) and expectations of favorable outcomes (outcome expectations). The use of resources (facilitation) and rewards (incentive motivation) also support behavior change. TTM behavioral strategies emphasize variation by stage of change including experiential processes during initial phases of behavioral adoption and behavioral processes during action and maintenance behavior change[49]. Consistent with the ongoing operation of Fair Oaks Clinic, VAFO emphasizes cultural congruence by providing the intervention entirely in Spanish via a bi-cultural staff that includes a CHW who is a member of the Fair Oaks community.

At the first CM and ES sessions, VAFO interventionists work with participants to identify their beliefs about the value of achieving healthy weight. The interventions emphasize that healthy weight is achievable through gradual and sustained lifestyle change that prioritizes nutrition and physical activity. Throughout the intervention CM and CHW interventionists remind participants to use fundamental outcome expectations (e.g. improved health and energy to enjoy family) as motivation for daily behavioral choices.

After establishing fundamental outcome expectations and a belief in self-efficacy for weight loss, the interventionists focus on knowledge and self-regulation to integrate new skills into daily behavior. At each intervention session participants learn about fundamental nutrition and physical activity skills such as balanced diet, portion control, diverse physical activities with a focus on walking, and shopping skills (Table 3). Application of SCT learning concepts includes educational materials and observing the interventionist perform activities. Additionally, group sessions employ experiential learning techniques including: preparing healthy foods, in-session physical activity, group problem solving and goal-setting, and role-playing with simulated or real-life scenarios for menu ordering, shopping, and portion sizes. CHW support sessions include the previously described techniques and include practicing new skills with the CHW in the home and community. Numerous materials are employed for observational and experiential learning at individual CM sessions.

**Table 3:** Required group and community health worker support session topics

Group session topics			
	
Session #	Physical activity	Nutrition	Educational incentive
1	Overview of healthy nutrition	Overview of exercise	Pedometer
2	Eating healthy on a budget	Being active at home	Fruit and vegetable guide
3	Eating out and fast food	Local exercise resources	Water bottle
4	Portion control and measuring	Hidden times for exercise	Measuring cups
5	Label reading and breakfast	Injury prevention and treatment	Massage tool
6	Healthy drinks	Building strong muscles	Healthy drink ingredients
7	Mindful eating and lunch	Take a deep breath and relax	Muscle relaxation CD
8	Healthy fast food and dinner	Exercise with friends and family	Whole grain pasta
9	Eating at social events and holidays	Mini holidays for exercise	Food pamphlet
10	Healthier traditions and review	Review and social dancing	Recipe with ingredients
11	Relapse response	Review and relay games	Motivational letter
12-15	Review relapse response, problem solving, and goal setting		Social support cards and displayable health guides

Community health worker support session topics*			
Nutrition			
Beverage inventory, evaluation of milk and water consumption, and goal for healthy beverages			
Evaluation of fat in cooking and goal for cooking with less fat (steaming, baking, etc.)			
Evaluation of fruit and vegetable consumption and goal for consuming more fruits and vegetables			
Evaluation of high calorie foods and goals for reduction of high calorie food or snacking			
Decision-making for shopping and meal planning			
Physical activity			
Chart and practice walking route (at first environmental support session)			
Identify support network of friends and family members for exercise			
Identify exercise locations in and around home			
Select physical activity goal			
Identify new physical activities to try			

*Topics may be covered in any order, but must be during the intensive intervention phase.			

CM and CHW interventionist use fundamental SCT self-regulation concepts to help participants translate knowledge about skills into behavior change. All intervention sessions teach participants goal-setting techniques for creating and maintaining specific, measurable, and obtainable goals with equal attention to nutrition and physical activity. Key self-monitoring techniques used at all intervention sessions include calendars and goals sheets to record health habits. Additionally, prior to the second CHW session, participants take pictures of meals and physical activities which are used to set "photo goals" and help evaluate accomplishments over the course of the study.

Key intervention feedback and reward techniques include: 1) self-reflection that acknowledges negative thoughts and counters them with positive statements; and 2) praise and positive reflection for achieving goals. Both interventions teach participants to prepare to overcome barriers (e.g. time commitments, family disagreements, economics, behavior relapses) by using problem solving and goal-setting skills. Group sessions use educational incentives such as take home tips sheets and health tools related to the session topic for motivation (Table 3). Later group sessions assume basic knowledge about topics from earlier sessions and increasingly focus on techniques to overcome barriers, maintain healthy behavior, respond to behavior relapse, and reach health goals. CHW sessions are loosely constructed to allow CHWs to address a range of behavioral goals, but must address several specific health topics (Table 3).

All intervention sessions promote collective efficacy and self-regulation by helping participants utilize social support networks. Group sessions facilitate the development of social support networks among group members by encouraging interaction outside of group sessions. At group sessions participants also develop collective efficacy and social support through "virtual walking groups." Individual steps are converted to collective miles traveled and used in a multi-media virtual travel adventure "Steps through the Americas" that presents health topics within the context of destinations in North and South America. Additionally, group and CHW sessions also promote social support by including family members and friends in session activities.

#### Intervention phases and session structure

Both CM and CM+CHW interventions involve intensive intervention (months 1-12) and maintenance (months 13-24) phases. In order to maintain focus on obesity reduction, VAFO interventionists follow a protocol to refer patients to other health care services (e.g., PCP, mental health, diabetes clinic) and community resources (e.g., health insurance programs, immigration assistance) for issues not directly related to weight loss.

#### Intervention phases

**Figure 1:**
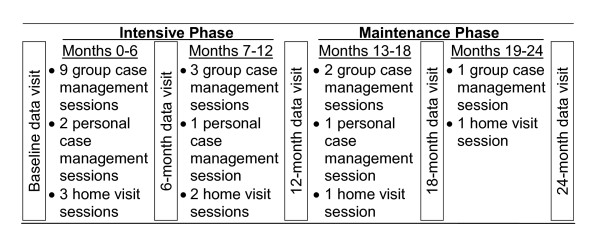
**Schedule of data collection and intervention visits**. All participants receive data collection visits. Case management visits are provided to participants in the case management and case management plus community health worker arms. Home visits are provided by community health workers to participants in the case management plus community health worker arm.

The intensive phase for both interventions includes a more intensive first six months and a less intensive second six months. The first six months includes nine group sessions and one individual CM session. The second six months includes three group sessions and two individual CM sessions. CM+CHW participants receive three CHW sessions during the first six months and two CHW sessions during the second six months. The maintenance phase (months 13-24) includes three group sessions and one individual CM session. CM+CHW participants also receive two CHW sessions (Figure 1).

#### Session structure

Each group session lasts two hours and follows a similar structure with four main components: 1) interactive ice-breaker; 2) experiential nutrition activity; 3) experiential physical activity; and 4) closing review and reminders. Individual CM sessions complement group session curriculum and CHW sessions by providing personal counseling for weight reduction goals. Individual sessions each last 30 minutes and include assessment of existing goals, problem solving for barriers and relapses, goal refinement, implementation for appropriate referrals, and evaluation of progress towards long-term goals. CHW sessions expand upon CM sessions with practical implementation of health goals within the home and community. VAFO CHWs are versed in group session curriculum, chronic disease management, and Fair Oaks neighborhood resources.

### Participant safety

After receiving PCP approval for the intervention, participants are screened for exclusion criteria and a Physical Activity Readiness Questionnaire is conducted[50]. Adverse Events Screening is administered at each data collection point to monitor for unexpected health events and the onset of new diseases. Staff follows a referral protocol for high blood pressure and PCPs review lab results for out-of-range values as part of their routine practice. Additionally, the study protocol is monitored by two IRBs and a Data and Safety Monitoring Board composed of two physicians and a PhD clinical trial epidemiologist not affiliated with any of the organizations involved in the study.

### Adherence and retention

VAFO uses four strategies to maximize participant adherence and retention: 1) careful eligibility screening; 2) staff-participant rapport building and motivational interviewing; 3) participant incentives at study visits; and 4) flexible scheduling. Quarterly newsletters are sent to encourage retention and capture address changes. Prior to dropping participants from the study, staff follows a protocol to offer modified status options, such as completing forms by phone or collecting weight measurement with a study scale at the participant's home.

### Sample size

Based on research literature and population trends in weight-gain, we hypothesize weight loss of 6% in CM+CHW arm and 2.5% in CM arm and weight gain of 2% in the UC arm at 24 months post-randomization. The study sample size provides adequate statistical power to test the primary hypothesis that CM+CHW will show greater reductions in BMI over 24 months than CM alone and, in turn, either intervention will produce greater reductions in BMI than UC. A sample size of 200 participants (80 in CM, 80 in CM + CHW, and 40 in UC), is estimated to provide 82% power for detecting 3.5% difference in BMI between the active treatments at alpha = 0.05 (two-sided) while allowing for 15% missing rate for the primary outcome. The sample size also provides greater than 79% power to detect 4.5% or greater difference in BMI between UC and either active treatment at alpha = 0.025.

### Data analysis

The primary hypothesis is that *patients managed through the CM+CHW intervention will show greater reductions in BMI over 24 months than those in CM*. We will model a random-effects regression on an intention-to-treat basis to assess the effects of intervention on BMI over 24 months. A hierarchical random effects model appropriately accounts for clustering of patients by PCP and multiple measurements on individuals over time. Using a randomized effects model, we will estimate the effect of intervention assignment on BMI while controlling for important covariates such as gender, baseline BMI, and diabetes status. All primary and secondary outcome analyses will use Holm's adjustments for multiple comparisons[51] which is more powerful than the Bonferroni method in adjusting for all pairwise comparisons[52] and for controlling the family-wise error rate.

Random-effects regression models will also be used to test secondary hypotheses: 1) *Patients in CM or CM+CHW intervention will experience reduced CHD risk through favorable changes in obesity-related risk factors relative to those in usual care*; 2) *Patients in the CM+CHW intervention will experience smaller increases in BMI from 12 months to 24 months than those in CM*; and 3) C*hange in BMI and other cardiovascular risk factors attributable to the intervention arm will be cost-effective relative to usual primary care*.

Estimation for the cost-effectiveness analysis will include: measurement of costs, measurement of changes in projected quality-adjusted life years, and calculation of a cost-effectiveness ratio for each active intervention compared to usual care. Health care costs associated with CHD over a 10-year period will be derived based on Framingham risk scoring[53,54] Interim analyses will be completed from 12-month data and when 24-month data is obtained from half of the sample, with appropriate adjustment for sequential examination of the data. Potential mediators (e.g., treatment adherence, caloric intake, physical activity level) and moderators (e.g., age, gender, country of origin, baseline mental status) of the intervention's effect on weight change also will be examined. Study participants will be analyzed as randomized (i.e., "intention to treat") regardless of subsequent intervention adherence. Alternative methods for handling missing data such as multiple imputations will be used for missing outcome data.

### Data management

The main study database uses research electronic data capture (REDCap) software that simultaneously receives data from multiple computers[55]. Additional databases include: 1) Omron BiLink database for pedometer data; 3) MS Access database for qualitative recruitment data; and 3) NutritionQuest Online database for Block Brief Food Questionnaire data. The data security protocol provides encryption, server-client authentication, and off-site backups to protect patient confidentiality and data integrity. Real-time data validation and weekly data quality reports will reduce data errors.

### Treatment fidelity

To ensure intervention fidelity, VAFO measures compliance and adherence to delivery of program components, the amount of the intervention received by participants, and delivery quality. VAFO staff follows standardized study protocol detailed in procedure manuals for individual counseling sessions, group health education classes, and home health visits. Following this protocol, VAFO staff prepares educational materials, incentives, and learning activities to deliver at specified visits. VAFO staff completes visit forms to record delivery and receipt of program components by participants. Forms and oral feedback from interventionists are reviewed weekly for completion. Deviation from the protocol is discussed at least weekly with senior researchers and aions are taken to keep in congruence with the study protocol. Prior to conducting visits, VAFO staff practices mock sessions and complete a training protocol with senior researchers. Additionally, 2% of intervention sessions are recorded for evaluation by senior staff to ensure that sessions are conducted according to protocol.

## Discussion and Conclusions

Health consequences of the obesity epidemic make it a primary health care priority. To this end, the USPSTF recommends that clinicians screen adults for obesity and offer intensive lifestyle counseling[56] However, clinical providers have limited training in such counseling and even greater impediments addressing social and environmental factors that influence the development of obesity[28-30]. Similarly, community-based organizations and health promoters acting to reduce environmental barriers are limited in their ability to respond to obesity-related co-morbidities. Coordinated efforts between clinic and community-based obesity interventions may be more effective than either approach alone. The need for congruent efforts is particularly important in low-income communities that experience disproportionate health disparities and environmental barriers[10]

VAFO is designed to test interventions that use intensive lifestyle counseling in clinical and community settings to confront obesity-related diseases and barriers in the built environment. Clinic-based health educators provide personal and group CM while community-based CHWs provide direct environmental support in participants' homes and neighborhoods. Health educators and CHWs are both trained to use SCT, TTM, and experiential learning concepts to promote healthy behaviors. VAFO health educators and CHWs both provide basic chronic disease management and facilitate patient utilization of primary care services and community resources. This novel study involves collaboration among academic, community and clinic partners with diverse expertise addressing the obesity epidemic ranging from primary care services and behavioral counseling to community-based exercise and nutrition classes and distribution of fresh fruits and vegetables through low-income food pantries.

Results from this study will provide valuable evidence about the efficacy and cost-effectiveness of two behavioral interventions to improve BMI, cardiovascular disease risk factors, and other psycho-social factors. VAFO will contribute knowledge about clinic-based lifestyle counseling and help discern the added benefit of a community-based behavioral intervention provided by CHWs. If proven efficacious, CHW support coordinated with clinic-based primary care CM may be a cost-effective and culturally sensitive way to extend evidence-based interventions for obesity reduction into low SES communities.

## Competing interests

RLD and JM declare no financial competing interests. Dr. Stafford reports a consulting relationship with Mylan Pharmaceuticals. Over the past five years, Dr. Stafford reports past honoraria from Bayer, and past grant funding through Stanford University from Procter and Gamble, Bayer, Merck and Company, SmithKlineGlaxo, Toyo Shinyaku, and Wako Chemical USA. All authors declare no non-financial competing interests.

## Authors' contributions

RSS and JM conceived of the study and RLD directed its design and implementation. RLD drafted the manuscript and RLD, JM, and RSS all critically reviewed the manuscript for important intellectual content. All authors read and approved the final manuscript.

## Acknowledgements and Funding

This study was funded by National Institutes of Health Grant HL089448. The content is solely the responsibility of the authors and does not necessarily represent the official views of the National Heart, Lung and Blood Institute or the National Institutes of Health. We are indebted to Gloria Flores-Garcia and Drs. Wes Alles, Jeanette Aviles, Christopher Gardner, William Haskell, Abby King, Donna Matheson, Marcia Stefanick, and Marilyn Winkleby for expert guidance on project development; to Dr. Lisa Goldman Rosas for services as project director; to Ernest Ceja, Rosa Gill, Priscilla Padilla, and Gabriela Spencer for services as intervention staff; to Oralia Espinoza, Alexis Fields, and Ulysses Rosas for research assistance and support; to Dr. Dave Ahn and Sreedevi Thiyagarajan for data management; to Diane Castle for assistance with management of project funds; to the Data and Safety Monitoring Board (Dr. Douglas Bauer [Chair], Sandra Bravo [Executive Secretary], Dr. Bud Gerstman, and Dr. Cecilia Gonzalez); to El Concilio of San Mateo County and the San Mateo Medical Center for collaboration and support; and to patients and their family for contribution to the research.

## Pre-publication history

The pre-publication history for this paper can be accessed here:

http://www.biomedcentral.com/1471-2458/11/98/prepub
